# Syms Operating Theatre

**Published:** 1890-03

**Authors:** 


Syms Operating Theatre.—Work has just been begun on the construction of the new building to be known as the William J. Syms Operating Theatre of Roosevelt Hospital, which the late Mr. Syms, a wealthy New York business man, who died last spring, willed $350,000 to construct and endow. The new building, as we have previously stated, will be of granite, and will occupy the present garden-plot on the Ninth avenue front of the grounds of Roosevelt Hospital. It will have a frontage of ninety feet on Fifty-nine street, and a depth of seventy-five feet, and will cost between $150,000 and $175,000, leaving nearly $200,000 for maintenance. The theatre will be for the free instruction in surgery of students and practitioners. The theatre proper will occupy the centre of the building, will rise to a height of three stories, and will have amphitheatre tiers of seats capable of accommodating three hundred persons. Around the amphitheatre will be built a one-story structure with plate-glass skylights, to be used for the manufacture of surgical dressings, implements, etc.—*Medical Record.*

---

An antiseptic ointment, certain in power and not unpleasant in odor, is often desired, not only by the obstetrician, but also by the gynæcologist. Dr. Th. Parvin says that benzoated lard to which 4 per cent. of creolin is added, will meet these indications satisfactorily.—*Texas Courier-Record.*

